# Supplementary figures and images for: The Arabian camel, Camelus dromedarius interferon epsilon: Functional expression, in vitro refolding, purification and cytotoxicity on breast cancer cell lines
Source: PLoS One. 2019 Sep 6;14(9):e0213880. doi: 10.1371/journal.pone.0213880 (PMC6730848; doi:10.1371/journal.pone.0213880)

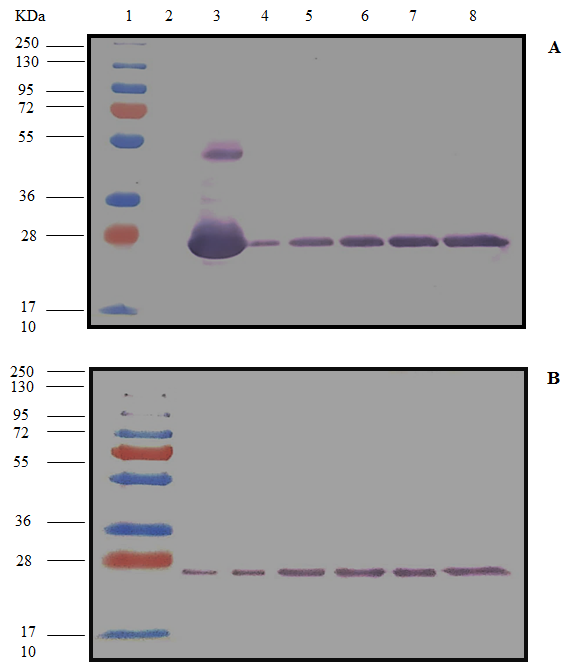

Supplement: S1 Fig — Western blotting analysis of C. dromedarius recombinant IFNε protein with 6x-His-Tag monoclonal antibody (1:1000 dilution). Panel (A): Lane 2 represents un-induced E. coli harboring pET28a(+) carrying the full-length cDNA; Lane 3, 50 μg of crude sonicated extract from lactose induced culture; Lanes 4–8 represent nickel-affinity purified recombinant protein at 5 to 25 μg concentration. Panel (B), Lanes 2–7 represent nickel affinity purified recombinant protein at 2 to 12 μg concentration. Lanes 1 Panel A and B represent pre-stained protein molecular weight markers. (TIF) [file pone.0213880.s001.tif]
