# Supplementary material for: The Arabian camel, Camelus dromedarius interferon epsilon: Functional expression, in vitro refolding, purification and cytotoxicity on breast cancer cell lines
Source: PLoS One. 2019 Sep 6;14(9):e0213880. doi: 10.1371/journal.pone.0213880 (PMC6730848; doi:10.1371/journal.pone.0213880)
Supplement: S1 Table — (DOCX) [file pone.0213880.s002.docx]

**S1 Table:** Detection of gram-negative bacterial endotoxins using LAL Chromogenic Endotoxin Quantitation Kit

**RESULTS**

| Ab at 405nm | Blank | Stand.0.1EU/ml | 0.5EU/ml | 0.25EU/ml | 1 EU/ml | **INFɛ** |
| --- | --- | --- | --- | --- | --- | --- |
| Rep.1 | 0.073 | 0.1215 | 0.2249 | 0.5194 | 0.8895 | **0.0909** |
| Rep.2 | 0.0813 | 0.1412 | 0.2717 | 0.6195 | 0.8719 | **0.0807** |
|  |  |  |  |  |  |  |
| Average | 0.077 | 0.13135 | 0.2483 | 0.56945 | 0.8807 | **0.0858** |
| Ab-blank |  | 0.05435 | 0.1713 | 0.49245 | 0.8037 | **0.0088** |

-Slope=0.8304

-Concentration of endotoxin in INFɛ sample (x)= 0.00757 EU/ml
